# Lcn2-derived Circular RNA (hsa_circ_0088732) Inhibits Cell Apoptosis and Promotes EMT in Glioma via the miR-661/RAB3D Axis

**DOI:** 10.3389/fonc.2020.00170

**Published:** 2020-02-21

**Authors:** Tao Jin, Mingfa Liu, Yan Liu, Yuanzhi Li, Zhennan Xu, Haoqi He, Jie Liu, Yuxuan Zhang, Yiquan Ke

**Affiliations:** ^1^The National Key Clinical Specialty, Guangdong Provincial Key Laboratory on Brain Function Repair and Regeneration, Department of Neurosurgery, The Engineering Technology Research Center of Education Ministry of China, Zhujiang Hospital, Southern Medical University, Guangzhou, China; ^2^Department of Neurosurgery, Shantou Central Hospital, Affiliated Shantou Hospital of Sun Yat-sen University, Shantou, China; ^3^Department of Neurosurgery, Affiliated Hengyang Hospital of Southern Medical University (Hengyang Central Hospital), Hengyang, China; ^4^Key Laboratory of Mental Health of the Ministry of Education, Guangdong-Hong Kong-Macao Greater Bay Area Center for Brain Science and Brain-Inspired Intelligence, Southern Medical University, Guangzhou, China

**Keywords:** glioma, EMT, hsa_circ_0088732, miR-661, RAB3D

## Abstract

**Background:** Glioma is the most common malignant tumor of the central nervous system, and often displays invasive growth. Recently, circular RNA (circRNA), which is a novel non-coding type of RNA, has been shown to play a vital role in glioma tumorigenesis. However, the functions and mechanism of lipocalin-2 (Lcn2)-derived circular RNA (hsa_circ_0088732) in glioma progression remain unclear.

**Methods:** We evaluated hsa_circ_0088732 expression by fluorescence *in situ* hybridization (FISH), Sanger sequencing, and PCR assays. Cell apoptosis was evaluated by flow cytometry and Hoechst 33258 staining. Transwell migration and invasion assays were performed to measure cell metastasis and viability. In addition, the target miRNA of hsa_circ_0088732 and the target gene of miR-661 were predicted by a bioinformatics analysis, and the interactions were verified by dual-luciferase reporter assays. RAB3D expression was analyzed by an immunochemistry assay, and E-cadherin, N-cadherin, and vimentin protein expression were examined by western blot assays. A mouse xenograft model was developed and used to analyze the effects of hsa_circ_0088732 on glioma growth *in vivo*.

**Results:** We verified that hsa_circ_0088732 is circular and highly expressed in glioma tissues. Knockdown of hsa_circ_0088732 induced glioma cell apoptosis and inhibited glioma cell migration, invasion, and epithelial-mesenchymal transition (EMT). We found that hsa_circ_0088732 negatively regulated miR-661 by targeting miR-661, and *RAB3D* was a target gene of miR-661. In addition, inhibition of miR-661 promoted glioma cell metastasis and suppressed cell apoptosis. Knockdown of *RAB3D* induced cell apoptosis and suppressed cell metastasis. Moreover, hsa_circ_0088732 accelerated glioma progression through its effects on the miR-661/RAB3D axis. Finally, results from a mouse xenograft model confirmed that knockdown of hsa_circ_0088732 induced miR-661 expression, resulting in suppression of RAB3D expression and inhibition of tumor growth *in vivo*.

**Conclusion:** We demonstrated that hsa_circ_0088732 facilitated glioma progression by sponging miR-661 to increase RAB3D expression. This study provides a theoretical basis for understanding the development and occurrence of glioma, as well as for the development of targeted drugs.

## Introduction

Glioma is caused by the cancerous transformation of glial cells in the brain and spinal cord (1, 2). When compared with other types of tumors, glioma has a poor prognosis, and the median patient survival time is ≤2 years (3). Furthermore, the incidence of glioma has been continuously increasing (4). Although surgery is the main method for treating glioma, the tumor tissue cannot be completely removed (5, 6). While radiotherapy and chemotherapy provide certain curative effects on glioma, various side effects associated with those treatments limit their therapeutic effect (7, 8). At present, glioblastoma is associated with a short survival time and a uniformly fatal outcome, irrespective of the treatment provided (9). Therefore, it is of great importance to study the mechanism for the occurrence and development of glioma, and identify new therapeutic targets and treatment strategies.

The development of glioma is a complex biological process that involves multiple mechanisms and factors (10, 11). Numerous tumor suppressor genes, oncogenes, and growth factors have been confirmed to be involved in glioma progression (12–14). Recent studies have proven that other types of biomolecules, such as circular RNAs (circRNAs), also play essential roles in this process (15, 16). CircRNAs are a class of single-stranded covalently closed circular non-coding RNAs (ncRNAs) with neither a 5′-terminal nor 3′-terminal poly A tail (17). CircRNAs cannot be degraded by RNAase enzymes due to their uniquely stable structure, and are thus highly conserved (18). Numerous studies have suggested circRNAs as potential biomarkers for use in tumor diagnosis and therapy, based on their stability and specificity of expression (19). In recent years, studies have suggested that circRNAs are closely associated with the occurrence and development of tumors (20, 21), such as oral cancers (22), bladder cancer (23), non-small cell lung cancer (24), and hepatocellular carcinoma (25). However, the expression and function of circRNAs in glioma have rarely been studied.

The recently discovered tumor biomarker lipocalin-2 (Lcn2) was initially found to be associated with iron absorption, antimicrobial activity, and epithelial cell differentiation (26, 27). Several studies have demonstrated that Lcn2 expression is increased in the presence of acute or chronic inflammation, as well as in cancer (28, 29). In addition, Lcn2 is capable of interacting with matrix metalloproteinases via the formation of complexes, and then participating in the cancer cell invasion process (30). Our previous studies showed that NGAL, coded by *Lcn2*, is associated with the clinical prognosis of glioma (31, 32). It is known that circRNA is formed by the variable splicing of mRNA (33). Currently, the underlying functions and mechanisms of Lcn2-derived circRNAs, and especially hsa_circ_0088732 in glioma, remain largely undetermined.

Recently, growing numbers of studies have reported that circRNAs can function as “miRNA sponges” and negatively regulate miRNAs (34, 35). Current studies have also confirmed that circRNAs can inhibit miRNA activity, and thus block the inhibitory effects of miRNAs on their target genes (36–38). MicroRNAs (miRNAs) are a type of highly conserved endogenous non-coding small RNA molecules consisting of 19–25 nucleotides, and directly regulate the levels of more plentiful mRNAs involved in different biological functions (39, 40). Increasing evidence suggests that miRNAs are abnormally expressed in glioma cells and involved in cell proliferation, differentiation, metabolism, apoptosis, and metastasis (41–43). However, the role played by hsa_circ_0088732 as an “miRNA sponge” in glioma has not been fully elucidated.

Rab GTPases are highly conserved intracellular transporter molecules, and basic components and major regulators of exocytic and endocytic membrane transport signaling pathways (44). RAB3D is one of the most important members of the Rab GTPase family, and an essential regulator of protein secretion. Within cancer cells, RAB3D activates intracellular AKT/GSK3β signaling to induce cell growth and metastasis (45). In the present study, we examined the expression levels of a novel circRNA (hsa_circ_0088732) in glioma tissues and cells, and also examined the function and mechanism of hsa_circ_0088732 in LN229 and U87-MG cells. In addition, we observed and analyzed the effects of hsa_circ_0088732 on miR-661, and proved that RAB3D is a direct target of miR-661. Taken together, our data indicate that hsa_circ_0088732 regulates RAB3D expression by targeting miR-661. Therefore, we for the first time suggest that the hsa_circ_0088732/miR-661/RAB3D axis may be a signaling pathway that can be of assistance in diagnosing and treating glioma.

## Materials and Methods

### Clinical Samples

Twenty pairs of glioma and adjacent non-tumor tissues [Normal, and located 2 cm from the contrast enhancement in a T1-weighted image's so-called clinical target volume (46)] were obtained from glioma patients who were treated at the Affiliated Shantou Hospital of Sun Yat-sen University (Shantou, Guangdong, P.R. China) between March 2017 and January 2018. All patients provided their written informed consent for sample collection prior to the operation. The protocol for this study was reviewed and approved by the Ethics Committee of the Affiliated Shantou Hospital of Sun Yat-sen University. The tissue biopsies were immediately stored at the −80°C. None of the patients enrolled in this study had received chemotherapy or radiotherapy prior to surgery. The patients were diagnosed and re-evaluated according to World Health Organization (WHO) criteria by two pathologists, and any differences of opinion were resolved by careful discussion.

### Fluorescence *in situ* Hybridization (FISH) Assay

The glioma tissues were fixed with 4% paraformaldehyde (Servicebio, China, G1113) for 6 h; after which, they were dehydrated in a graded ethanol series and embedded with paraffin (Sakura, Japan). After being sliced into sections (4 μm thick), the embedded tissues were incubated at 62°C for 2 h. Next, the sections were sequentially treated with dimethylbenzene xylene for 15 min, dimethylbenzene xylene for 15 min, anhydrous ethanol for 5 min, 85% alcohol for 5 min, and 75% alcohol for 5 min. The slide-mounted tissue sections were then treated with 3% H_2_O_2_ and proteinase K (2 μg/mL, Servicebio, G3016-1) at 37°C for 30 min, washed, pre-hybridized at 37°C for 1 h, and finally hybridized overnight at 46°C with 1 μL of hybrid solution that contained hsa_circ_0088732 probes (GenePharma, Shanghai, China). After washing, the slides were treated with 4′6-diamidino-2-phenylindole (DAPI, cat. no. 28718-90-3) solution for 8 min, and then visualized with a fluorescence microscope.

### Cell Culture

Normal HEB glial cells, 293T cells, and glioma cell lines LN229, U87-MG, U251, and A172 were obtained from the Type Culture Collection of the Chinese Academy of Sciences (Shanghai, China). The 293T, HEB, LN229, U87-M, and A172 cells were cultured in Dulbecco's Modified Eagle's Medium (DMEM, cat # 11965-118), and the U251 cells were cultured in RPMI 1640 medium (ATCC, cat #: 30-2001). All culture media were supplemented with 10% fetal bovine serum (FBS, cat # SH30071.03), 1% penicillin/streptomycin, and 2 mM glutamine. All the cells were grown at 37°C in a humidified atmosphere containing 5% CO_2_.

### RNA Interference and miRNA Transfection

The small interfering RNAs (siRNAs) mixture targeting hsa_circ_0088732 and *RAB3D*, as well as a negative control (NC), miR-661 mimics, and miR-661 inhibitors were purchased from GenePharma Co., Ltd. (Shanghai, China). LN229 and U87-MG cells were seeded into 6-well plates (1 × 10^5^/cells per well) and transfected with 10 nM NC, 10 nM hsa_circ_0088732 siRNAs and 10 nM RAB3D siRNAs or 10 nM miR-661 mimics, 10 nM miR-661 inhibitors and 10 nM control by using Lipofectamine® 2000 (Invitrogen) according to the manufacturer's protocol.

### Plasmid Construction and Transfection

hsa_circ_0088732 and *RAB3D* were amplified by using 2 × Phanta Max Buffer, dNTP Mix (10 mM each), and Phanta Max Super-Fidelity DNA Polymerase. The PCR products were recycled with a Gel Extraction kit (Omega Bio-tek, Norcross, GA, USA), and then inserted into a psiCHECK-2 vector (Promega, Madison, WI, USA Cat Number C8021). The primers for hsa_circ_0088732 consisted of a forward primer containing an XhoI site: 5′-CCGCTCGAGGGAGAACCAAGGAGCTGACTTCG-3′, and a reverse primer containing a NotI site: 5′-ATTTGCGGCCGCGGCCTGAGGGCACATGTTTATTTAG-3′. The primers for RAB3D consisted of a forward primer containing a Kpn site: 5′-CCGCTCGAGTGGAACTATGGACCACATTAGACTG-3′, and a reverse primer containing an XhoI site: 5′-ATTTGCGGCCGCGACAAGGATTGGGAAATGGACA-3′. LN229 and U87-MG cells were seeded into 6-well plates (1 × 10^5^ cells/well) and transfected with the hsa_circ_0088732-expression vector. The RAB3D-expression vector and control (pcDNA3.0) were transfected into cells by using Lipofectamine 3000 (Cat. No. L3000015) according to the manufacturer's protocol.

### RNA Extraction and Quantitative Real-Time PCR (RT-PCR)

Total RNA was extracted from glioma cells and tissues by using TRIzol reagent (Invitrogen, Carlsbad, CA, USA). A NanoDrop2000c system (Thermo Fisher Scientific, Waltham, USA) was used to evaluate the concentrations of various RNAs, and a First Strand cDNA Synthesis Kit (Thermo Fisher) was used to produce cDNA by reverse transcription. PCR assays were performed by using SYBR GREEN PCR Master Mix (Takala) on an ABI7500 Real-time PCR system (Applied Biosystems, Foster City, CA, USA). The sequences of the primers used are shown in Table 1. The relative levels of mRNA or miRNA were measured by the 2^−ΔΔ*Ct*^ method, and normalized to those for GAPDH or U6, respectively.

**Table 1:** The sequences of primers used in real-time PCR.

**Gene**	**Sequence(5′ - 3′)**
GAPDH F	TGTTCGTCATGGGTGTGAAC
GAPDH R	ATGGCATGGACTGTGGTCAT
hsa_circ_0088732 F	ATAAACATGTGCCCTCAGGC
hsa_circ_0088732 R	TTGGGACAGGGAAGACGATG
U6 F	CTCGCTTCGGCAGCACA
U6 R	AACGCTTCACGAATTTGCGT
All R	CTCAACTGGTGTCGTGGA
Hsa-miR-661	TGCCTGGGTCTCTGGCCTGCGCGT
Hsa-miR-661 RT	CTCAACTGGTGTCGTGGAGTCGGCAATTCAGTTGAGACGCGCA
Hsa-miR-661 F	ACACTCCAGCTGGGTGCCTGGGTCTCTGGCCTGC
Hsa-miR-7	TGGAAGACTAGTGATTTTGTTGTT
Hsa-miR-7 RT	CTCAACTGGTGTCGTGGAGTCGGCAATTCAGTTGAGAACAACA
Hsa-miR-7 F	ACACTCCAGCTGGGTGGAAGACTAGTGATTTTGT

*F, forward primer; R, reversed primer; RT, reverse transcription primer*.

### Polymerase Chain Reaction (PCR) Assay

The cDNA template (5 ng) was mixed with Ex Taq DNA Polymerase (1.25 U), the upstream primer (0.2 μM), the downstream primer (0.2 μM), dNTPs (4 μL, 1 mM each), and 10 × Ex Taq buffer (5 μL) according to the manufacturer's instructions. The reaction conditions were 30 cycles of 94°C for 4 min, followed by 94°C for 40 s, 65°C for 30 s, and 72°C for 1 min, and then by 72°C for 5 min. The PCR products were assessed by electrophoresis with a 1.0% agarose gel.

### Sanger Sequencing

The amplification product of the hsa_circ_0088732 sequence, including the splice sites, was verified by Sangon Biotech (Shanghai, China).

### Western Blot Assays

RIPA lysis buffer was used to extract the total proteins from treated glioma cells, and the protein concentration in each extract was quantified using a BCA protein assay kit (Amresco, Fountain Parkway Solon, OH, USA, FA016-50G). An aliquot of total protein from each treatment group was separated by 10% SDS-PAGE, and the protein bands were transferred onto polyvinylidene fluoride (PVDF) membranes (Millipore, Burlington, MA, USA, IPVH00010). The membranes were subsequently blocked with non-fat milk and then incubated with primary antibodies against RAB3D (1:10,000, Abcam, Cambridge, UK, ab128997), E-cadherin (1:1,000; Abcam, ab76055), N-cadherin (1:500; Abcam, ab18203), vimentin (1:500; Abcam, ab137321) or GAPDH (1:2,000; Abcam, ab8245) for 1 h at room temperature. After washing, the membranes were incubated with an HRP-conjugated secondary antibody (1:20,000; BOSTER, Pleasanton, CA, USA, BA1054) for 40 min. The immunostained proteins were visualized by using ECL reagent (Applygen Technologies, Beijing, China) and X-ray film (SUPER RX-N-C; Fuji, Japan).

### Transwell Assays

Transfected LN229 and U87-MG cells were harvested and counted. Next, 200 μL of cells (1 × 10^5^ cells/mL) were seeded into the upper chamber of a Transwell plate (8 μm pore size, Costor, Cat. No. 3422), and 500 μL of culture medium containing 15% FBS was added to the lower chamber. After incubation for 24 h at 37°C, the migrated cells were fixed with 4% paraformaldehyde for 20 min and then stained with 0.5% crystal violet (Beyotime Institute of Biotechnology, China) for 5 min. After washing, the cells in the upper chamber were removed, and the migrated cells were observed under a microscope (OLYMPUS CX41). Transwell plates used for invasion assays had their upper chambers coated with Matrigel 30 min prior to being used for assays.

### Cell Apoptosis Detection

#### Flow Cytometry Detection

The treated cells were harvested and counted, and then centrifuged at 1,000 g for 5 min; after which, they were washed and then stained for 15 min with reagents contained in an Annexin V-FITC Apoptosis Detection Kit (A211-01). Finally, the results for apoptosis were obtained by flow cytometry (FACSCalibur, BD, Franklin Lakes, NJ, USA).

#### Hoechst 33258 Staining

The treated cells were stained using Hoechst 33258 (Sigma-Aldrich, St. Louis, MO, USA) as recommended by the manufacturer. The morphology of the cell nucleus was observed using a fluorescence microscope (Olympus Corporation, Japan).

### Immunohistochemistry Assays

Paraffin embedded tissue sections (5 μm thick) were dewaxed with pure xylene and then rehydrated in a series of ethanol solutions. The sections were then blocked with serum and incubated with anti-RAB3D antibody (Abcam, ab3337) at 4°C overnight; after which, they were incubated with goat anti-rabbit serum for 20 min. Following incubation, the sections were treated with diaminobenzidine (DAB, Sigma Aldrich, Cat# 5637), and then counterstained with hematoxylin. Images of the stained tissues were analyzed by microscopy, and results are expressed as a staining intensity and positive rate.

### Dual-Luciferase Reporter Assays

The wild type (WT) and mutant (Mut) fragments of hsa_circ_0088732 and the 3′untranslated region (3′UTR) of RAB3D were amplified by PCR and inserted into the psiCHECK2 vector (Promega). The primer used for WT-hsa_circ_0088732 consisted of a forward primer containing an XhoI site: 5′-CCGCTCGAGGGAGAACCAAGGAGCTGACTTCG-3′, and a reverse primer containing a NotI site: 5′-ATTTGCGGCCGCGGCCTGAGGGCACATGTTTATTTAG-3′. The primers used for MUT-hsa_circ_0088732 were 5′-CCCATGCAGCTGCTCTGATTAGCACCCCGCTGATGGA-3′ (forward) and 5′-TCCATCAGCGGGGTGCTAATCAGAGCAGCTGCATGGG-3′ (reverse). The primers used for 3′UTR-RAB3D-WT consisted of a forward primer containing an XhoI site: 5′-CCGCTCGAGTGAAACTGACATCTTCTCAAATCTT-3′, and a reverse primer containing a NotI site: 5′-ATTTGCGGCCGCTATAGCCCTACACTGGAGGTCAA-3′. The primers used for 3′UTR-RAB3D-MUT were 5′-GTCCCCCTGCAGGTCTAACTCAAGCAGACAATTCCAC-3′ (forward) and 5′-GTGGAATTGTCTGCTTGAGTTAGACCTGCAGGGGGAC-3′ (reverse). The 293T cells were seeded into 96-well plates (1 × 10^4^ cells/well) and incubated at 37°C. The next day, the cells were co-transfected with miR-661 mimics plus WT-hsa_circ_0088732 or MUT-hsa_circ_0088732 or 3′UTR-RAB3D-WT or 3′UTR-RAB3D-MUT. The transfection rates determined by flow cytometry were >90%. Next, luciferase activity was assessed by using the Dual-Luciferase Assay System (Promega) according to manufacturer's instructions. Firefly luciferase activity was normalized to that of Renilla luciferase activity.

### The Xenograft Model

Male BALB/c Nude mice (*n* = 24; age = 5 weeks) were purchased from Charles River (Beijing, China). Treated LN229 cells in log phase growth were digested with trypsin and collected. After adjusting the cell density to 1 × 10^7^ cells/mL, 0.1 mL of digested cells (~1 × 10^6^ cells) was subcutaneously injected into the right axilla of each nude mouse. Tumor formation was examined every 3 days after injection. The mice were euthanized on day 21, and the tumor volumes were calculated by using the following modified ellipsoid formula: (L × W × W)/2, L: length, W: width. Tumor growth curves were drawn at the end of the experiment.

### Hematoxylin-Eosin (H&E) Staining

The heterotransplanted tumors were fixed in 4% paraformaldehyde solution and embedded in paraffin; after which, 4 μm sections were cut and stained with hematoxylin and eosin (H&E). The sections were examined under a light microscope at × 200 magnification.

### Immunohistochemistry (IHC)

Tissues were fixed in 4% paraformaldehyde solution and embedded in paraffin; after which, 4 μm sections were cut and immunostained. The slide-mounted sections were first incubated with Ki-67 primary antibody (1:100, Abcam, ab15580) for 1 h at 37°C, and then incubated with an HRP-conjugated secondary antibody for 60 min at room temperature. The sections were then counterstained with hematoxylin for 5 min, and images were collected under a microscope (Olympus, Tokyo, Japan) at × 200 magnification.

### Terminal Deoxynucleotidyl Transferase-Mediated dUTP Nick End-Labeling (TUNEL) Assay

TUNEL assays were performed to assess cell death. The assays were conducting using an *in situ* cell death detection kit (Roche Applied Science, Indianapolis, IN, USA) according to the manufacturer's instructions.

### Statistical Analysis

Each experiment was repeated three times, and results were expressed as the mean ± SD. All data were analyzed using Graphpad Prism software, Ver. 7 (GraphPad Prism Software, La Jolla, CA, USA). One-way analysis of variance was used to assess the significance of differences between different groups; the correlation between hsa_circ_0088732 and miR-661 was analyzed by Pearson's correlation coefficient. A *P*-value <0.05 was considered to be statistically significant.

## Results

### Identification of Lcn2-derived circRNAs

**Figure 1:**
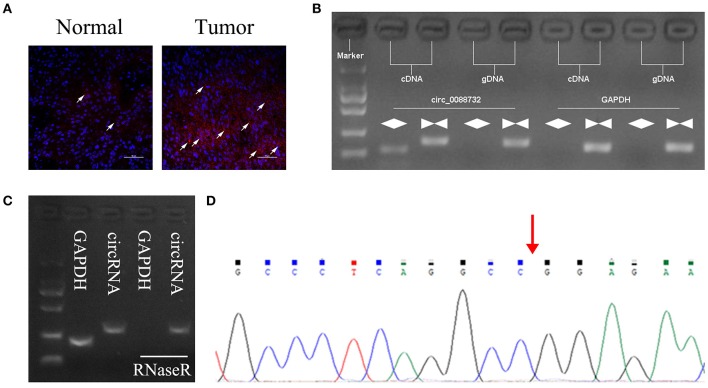
Identification of hsa_circ_0088732. **(A)** Fluorescence *in situ* hybridization (FISH) was performed to show the levels and locations of Lcn2-derived circRNA (hsa_circ_0088732) expression. Red indicates circRNA and blue indicates nuclei (DAPI). The white arrow indicates hsa_circ_0088732. Magnification, ×200; Scale bar = 100 μm. **(B)** The linear and circular Lcn2 in cDNA and gDNA were amplified by using convergent and divergent primers, respectively. **(C)** qRT-PCR was used to analyze the expression of GAPDH and hsa_circ_0088732 RNA after treatment with RNaseR. **(D)** Sanger sequencing was used to verify the circular structure of hsa_circ_0088732; red arrow shows the site of the backsplice junction.

According to the circbase and circNet databases, Lcn2 can form hsa_circ_0088732 by cyclization. To explore the role of Lcn2-derived circRNA (hsa_circ_0088732) in glioma, a FISH probe was designed and used to examine the levels and locations of hsa_circ_0088732 expression in glioma tissues. The results showed that hsa_circ_0088732 was highly expressed in glioma tissues, and mainly located in the cytoplasm (Figure 1A; the white arrow indicates hsa_circ_0088732 expression). To verify the formation of hsa_circ_0088732, we designed convergent primers and divergent primers that could amplify the reference gene (GAPDH) and circLcn2 (hsa_circ_0088732) by using cDNA and gDNA as templates. The results revealed that hsa_circ_0088732 could be amplified by divergent cDNA primers, and no products were observed in the gDNA groups, suggesting that hsa_circ_0088732 could be formatted (Figure 1B). Furthermore, we found that the PCR products of GAPDH were not observed in cDNA after treatment with RNaseR, but hsa_circ_0088732 could be amplified (Figure 1C). In addition, we used Sanger sequencing to verify the circular structure, and found that the sequence was in accordance with hsa_circ_0088732 (Figure 1D; red arrow shows the site of the backsplice junction).

### hsa_circ_0088732 Regulated Glioma Cell Apoptosis

**Figure 2:**
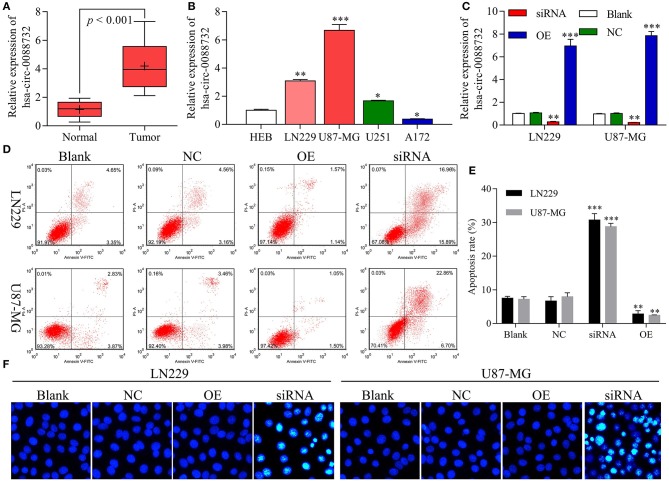
hsa_circ_0088732 regulated glioma cell apoptosis. **(A)** qRT-PCR assays were performed to detect the expression of hsa_circ_0088732 in 20 pairs of glioma and adjacent non-tumor tissues (*P* < 0.001). **(B)** qRT-PCR assays were performed to assess the expression of hsa_circ_0088732 in HEB and glioma cells, including LN229, U87-MG, U251, and A172 cells (^*^P < 0.05, ^**^P < 0.01, ^***^P < 0.001). **(C)** Knockdown or overexpression of hsa_circ_0088732 in LN229 and U87-MG cells was determined by qRT-PCR assays (^**^P < 0.01). **(D–F)** PI/Annexin V FITC and Hoechst 33258 staining were used to evaluate cell apoptosis (^**^P < 0.01). NC: negative control, siRNA: hsa_circ_0088732 siRNAs, OE: hsa_circ_0088732 overexpression plasmids.

Our results revealed that hsa_circ_0088732 was significantly upregulated in samples of glioma tissue when compared to samples of adjacent non-tumor tissue (*P* < 0.001, Figure 2A). We also found that hsa_circ_0088732 expression was significantly increased in glioma cells (LN229, U87-MG, and U251) when compared with HEB cells (*P* < 0.05, *P* < 0.01, *P* < 0.001, Figure 2B). Because hsa_circ_0088732 was highly expressed in glioma, the LN229 and U87-MG cell lines were used for further studies. To further investigate the biological functions and molecular mechanisms of hsa_circ_0088732 in glioma, hsa_circ_0088732 siRNA and overexpression plasmids were used to transfect LN229 and U87-MG cells. The qRT-PCR assay was used to determine the efficiency of hsa_circ_0088732 knockdown, and the results indicated that hsa_circ_0088732 expression was significantly decreased in the siRNA group relative to the control group, suggesting that hsa_circ_0088732 expression had been effectively blocked. Furthermore, hsa_circ_0088732 expression was significantly increased in the overexpression (OE) groups (*P* < 0.01, Figure 2C). Flow cytometry and Hoechst 33258 staining results showed that cell apoptosis was markedly increased in the siRNA group when compared with the blank group, and significantly decreased in the OE group when compared with the blank group. Therefore, these results demonstrated that knockdown of hsa_circ_0088732 could promote cell apoptosis, and overexpression of hsa_circ_0088732 could inhibit apoptosis in glioma cell lines (*P* < 0.01, Figures 2D–F).

### hsa_circ_0088732 Regulated Glioma Cell Migration, Invasion, and Epithelial-Mesenchymal Transition (EMT)

**Figure 3:**
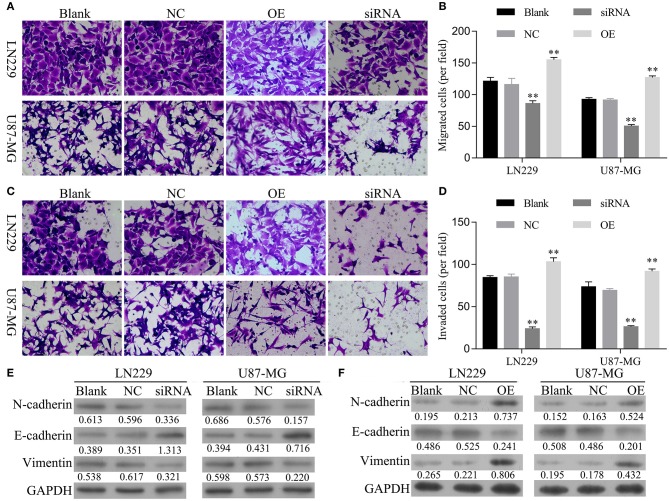
hsa_circ_0088732 regulated glioma cell migration, invasion, and epithelial-mesenchymal transition (EMT). **(A–D)** The effect of hsa_circ_0088732 knockdown or overexpression on cell migration and invasion was evaluated by Transwell assays. **(E,F)** The expression of EMT-related proteins (E-cadherin, N-cadherin, and vimentin) was examined by western blotting. NC: negative control, siRNA: hsa_circ_0088732 siRNAs, OE: hsa_circ_0088732 overexpression plasmids.

Next, the effects of hsa_circ_0088732 on glioma cell migration and invasion were assessed by Transwell assays. First, we analyzed the migration and invasion abilities of different glioma cell lines, and found that both abilities were positively correlated with levels of hsa_circ_0088732 expression (Figure S1). We also found that overexpression of hsa_circ_0088732 promoted HEB and A172 cell migration and invasion (Figure S2). Our results revealed that the numbers of migrated and invaded cells were significantly reduced in the siRNA group and markedly increased in OE group when compared with the blank group (*P* < 0.05, Figures 3A–D). In addition, the western blot results showed that knockdown of hsa_circ_0088732 downregulated N-cadherin and vimentin expression and upregulated E-cadherin expression, while overexpression of hsa_circ_0088732 induced N-cadherin and vimentin expression and reduced E-cadherin expression, suggesting that knockdown or overexpression of hsa_circ_0088732 inhibited or promoted glioma cell EMT, separately (Figures 3E,F).

### hsa_circ_0088732 Sponged miR-661 and Negatively Regulated miR-661 Expression

**Figure 4:**
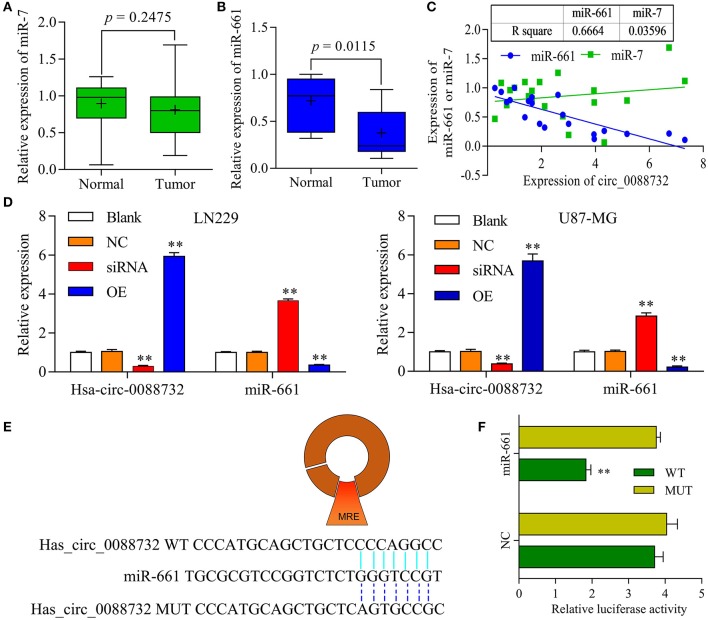
hsa_circ_0088732 sponged miR-661 and negatively regulated miR-661 expression. **(A,B)** The levels of miR-7 and miR-661 expression in 20 pairs of glioma and adjacent non-tumor tissues were analyzed by qRT-PCR. **(C)** Person's correlation coefficient was used to analyze the correlation between hsa_circ_0088732 and miR-7 (R^2^ = 0.03596) and miR-661 (R^2^ = 0.6664). **(D)** LN229 and U87-MG cells were transfected with hsa_circ_0088732 siRNAs or overexpression plasmids, respectively. hsa_circ_0088732 and miR-661 expression were analyzed by qRT-PCR assays (^**^P < 0.01). **(E)** The potential targets of hsa_circ_0088732 were analyzed by a bioinformatics analysis, and a binding site between hsa_circ_0088732 and miR-661 was identified. **(F)** 293T cells were co-transfected with WT-hsa_circ_0088732 or MUT-hsa_circ_0088732, and with miR-661 or the NC, and the relative levels of luciferase activity were analyzed (^**^P < 0.01). NC: negative control, siRNA: hsa_circ_0088732 siRNAs, OE: hsa_circ_0088732 overexpression plasmids.

To explore the underlying molecular mechanisms of hsa_circ_0088732 in glioma, a bioinformatics analysis was performed using the public database CircInteractome (https://circinteractome.nia.nih.gov/) to identify the target miRNAs of hsa_circ_0088732. We found that current publications had reported that miR-661 was clinically associated with glioma. Next, we performed qRT-PCR assays to identify the levels of miR-7 and miR-661 in glioma tissues, and found a decrease in miR-661 levels (*P* = 0.0115) but no change in miR-7 levels (*P* = 0.2475) in glioma tissues when compared with adjacent non-tumor tissues (Figures 4A,B). In addition, we analyzed the correlation of hsa_circ_0088732 with miR-7 and miR-661. The results showed that hsa_circ_0088732 displayed a significant negative correlation with miR-661, but not with miR-7 (Figure 4C). To further explore the regulatory effect of hsa_circ_0088732 on miR-661, LN229 and U87-MG cells were transfected with hsa_circ_0088732 siRNA or overexpression plasmids. Results from qRT-PCR assays revealed that knockdown of hsa_circ_0088732 promoted miR-661 expression, and overexpression of hsa_circ_0088732 inhibited the expression of miR-661 (*P* < 0.01, Figure 4D). In addition, a bioinformatics analysis performed to predict and screen miRNAs that might be targeted by hsa_circ_0088732 showed that a hsa_circ_0088732 binding site existed for miR-661 (Figure 4E). The results also showed that there was a decreased luciferase intensity between the WT-hsa_circ_0088732 and miR-661, while there was no difference between the luciferase intensities of MUT-hsa_circ_0088732 and miR-661 (*P* < 0.01, Figure 4F). Our results confirmed that hsa_circ_0088732 could serve as a sponge to directly regulate miR-661 expression.

### The Restoration of miR-661 Inhibition Led to Inhibition of Apoptosis and Promoted Glioma Cell Migration, Invasion, and EMT Mediated by hsa_circ_0088732 Knockdown

**Figure 5:**
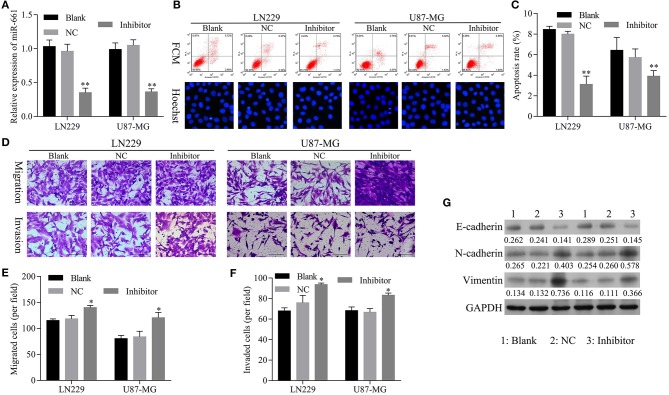
Transfection with an miR-661 inhibitor suppressed glioma cell apoptosis and induced cell migration and invasion. **(A)** qRT-PCR assays were performed to detect miR-661 expression. **(B,C)** Flow cytometry and Hoechst 33258 staining were used to detect cell apoptosis. **(D–F)** The effect of miR-661 knockdown on cell migration and invasion was evaluated by Transwell assays. **(G)** The expression of EMT-related proteins (E-cadherin, N-cadherin, and vimentin) was examined by western blot assays (^*^P < 0.05, ^**^P < 0.01). NC: negative control, Inhibitor: miR-661 inhibitor.

**Figure 6:**
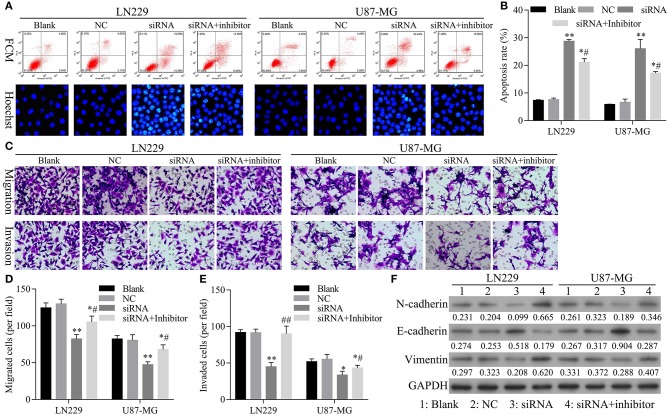
The restoration of miR-661 inhibition led to inhibition of apoptosis, and promoted glioma cell migration, invasion, and EMT mediated by hsa_circ_0088732 knockdown. LN229 and U87-MG cells were transfected with hsa_circ_0088732 siRNAs and/or an miR-661 inhibitor, respectively. **(A,B)** Cell apoptosis was detected by PI/Annexin V FITC and Hoechst 33258 staining (^*^P < 0.05, ^**^P < 0.01 vs. NC group; ^#^*P* < 0.05 vs. siRNA group). **(C–E)** Cell migration and invasion capabilities were evaluated by Transwell assays. **(F)** The levels of E-cadherin, N-cadherin, and vimentin expression were determined by western blotting. NC: negative control, siRNA: hsa_circ_0088732 siRNAs, Inhibitor: miR-661 inhibitor.

To confirm that knockdown of miR-661 could restore the inhibition of apoptosis and promotion of glioma cell migration, invasion, and EMT mediated by hsa_circ_0088732 knockdown, we first verified that transfection of an miR-661 inhibitor could inhibit LN229 and U87-MG cell apoptosis and promote the migration and invasion capabilities of LN229 and U87-MG cells by regulating N-cadherin, vimentin, and E-cadherin expression (Figure 5). Furthermore, we investigated the effects of hsa_circ_0088732 knockdown on glioma cell migration and invasion by suppressing miR-661. LN229 and U87-MG cells were transfected with hsa_circ_0088732 siRNA and/or an miR-661 inhibitor, respectively. Our data showed that the miR-661 inhibitor could attenuate the increase in glioma cell apoptosis induced by hsa_circ_0088732 knockdown (*P* < 0.05, *P* < 0.01, Figures 6A,B). Results from Transwell assays showed that cellular migration and invasion capabilities were significantly enhanced in the hsa_circ_0088732 siRNA+miR-661 inhibitor group when compared with the hsa_circ_0088732 siRNA group, indicating that an miR-661 inhibitor could reverse the inhibitory effect of hsa_circ_0088732 knockdown on glioma cell migration and invasion (*P* < 0.05, Figures 6C–E). Our results also revealed that N-cadherin and vimentin expression levels were increased and E-cadherin expression was decreased in the hsa_circ_0088732 siRNA+miR-661 inhibitor group relative to the hsa_circ_0088732 siRNA group (Figure 6F). These results suggested that knockdown of miR-661 could restore the inhibition of apoptosis and promotion of glioma cell migration, invasion, and EMT process mediated by hsa_circ_0088732 knockdown.

### RAB3D Served as a Target of miR-661

**Figure 7:**
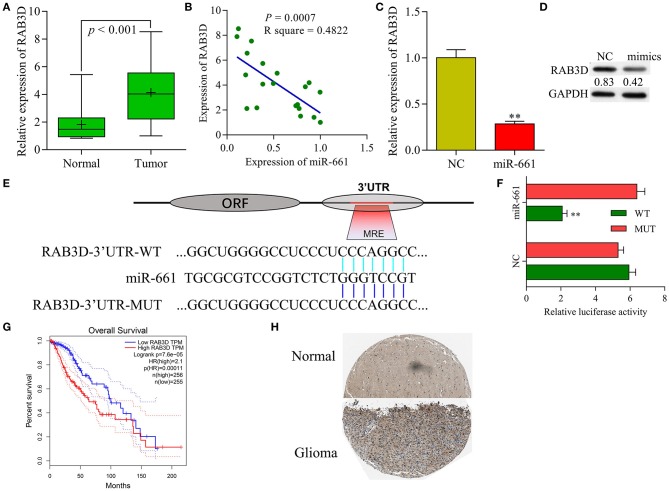
RAB3D served as a target of miR-661. **(A)** RAB3D mRNA expression in samples of normal and tumor tissues was examined by qRT-PCR. **(B)** Person's correlation coefficient was used to analyze the correlation between RAB3D and miR-661. **(C,D)** RAB3D expression was measured by qRT-PCR and western blot assays after transfection with miR-661 mimics (^**^P < 0.01). **(E)** The binding site between RAB3D and miR-661 was identified. **(F)** 293T cells were co-transfected with WT-RAB3D or MUT-RAB3D and miR-661 or NC; dual luciferase reporter assays were used to analyze relative luciferase activity. **(G)** GEPIA showing the overall survival of glioma patients. **(H)** RAB3D expression in glioma and adjacent non-tumor tissues was assessed by immunochemistry (online database: The human protein atlas).

Our qRT-PCR results showed that RAB3D expression was significantly upregulated in samples of glioma tissue when compared with samples of adjacent non-tumor tissue (Figure 7A), and that levels of RAB3D expression were negatively correlated with those of miR-661 expression (Figure 7B). Meanwhile, results of immunochemistry assays obtained from the public database The Human Protein Atlas (https://www.proteinatlas.org/) showed that RAB3D was expressed at much higher levels in glioma tissues than in adjacent non-tumor tissues (*P* < 0.05, Figure 7H). qRT-PCR and western blot results showed that overexpression miR-661 significantly suppressed the expression of RAB3D (Figures 7C,D). In addition, we used TargetScan, miRDB, and microorna.org to identify a binding site for miR-661 on RAB3D mRNA (Figure 7E), and the results of dual-luciferase reporter assays indicated that miR-661 significantly decreased the luciferase intensity of WT-RAB3D, while miR-661 had no effect on the MUT-RAB3D (*P* < 0.05, Figure 7F). Moreover, a search of information in Gene Expression Profiling Interactive Analysis (GEPIA, http://gepia.cancer-pku.cn/index.html) revealed that glioma patients with tumors that displayed high levels of RAB3D expression had shorter survival times (*P* = 0.0001), suggesting the relevance of RAB3D to the survival of glioma patients (Figure 7G). Taken together, our results suggest that *RAB3D* is a target gene of miR-661.

### Knockdown of RAB3D Facilitated Glioma Cell Apoptosis and Inhibited Glioma Cell Migration, Invasion, and EMT

**Figure 8:**
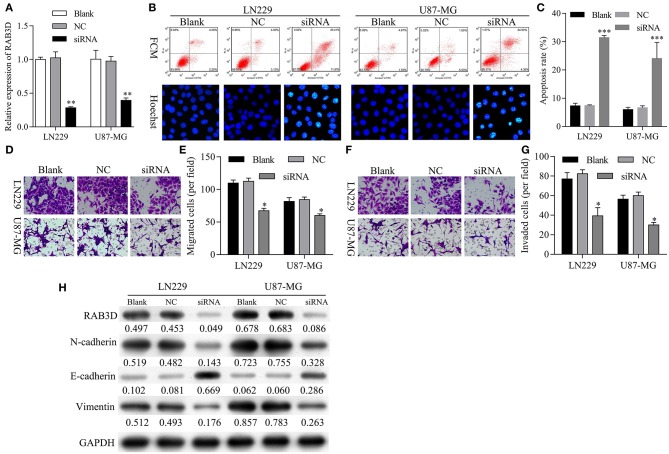
Knockdown of RAB3D facilitated glioma cell apoptosis and inhibited glioma cell migration, invasion, and EMT. **(A)** RAB3D expression in LN229 and U87-MG cells was inhibited by transfection with RAB3D siRNA, and the efficiency of RAB3D knockdown was evaluated by qRT-PCR assays (^*^P < 0.05, ^**^P < 0.01, ^***^P < 0.001). **(B,C)** Cell apoptosis in RAB3D-silenced LN229 and U87-MG cells was examined by flow cytometry and Hoechst 33258 staining. **(D–G)** The migration and invasion capabilities of LN229 and U87-MG cells with RAB3D knockdown were assessed by Transwell assays. **(H)** The levels of E-cadherin, N-cadherin, and vimentin were examined by western blotting. NC: negative control, siRNA: RAB3D siRNA.

Next, we further investigated the effects of RAB3D on glioma progression. To assess the biological functions of RAB3D, specific siRNAs against RAB3D were synthesized and used to downregulate RAB3D expression in LN229 and U87-MG cells. The results showed that the RAB3D siRNAs could effectively knock down RAB3D expression (*P* < 0.01, Figure 8A). Flow cytometry and Hoechst 33258 staining results showed a significant promotion of cell apoptosis among RAB3D-silenced LN229 and U87-MG cells (*P* < 0.01, Figures 8B,C). Transwell assays showed that the migration and invasion capabilities of LN229 and U87-MG glioma cells transfected with RAB3D siRNA were significantly reduced when compared to those capabilities for control cells (*P* < 0.05, Figures 8D–G). Moreover, we demonstrated that after RAB3D knockdown, E-cadherin expression was upregulated, while N-cadherin and vimentin expression were downregulated (Figure 8H). These data indicated that knockdown of *RAB3D* could promote glioma cell apoptosis, and inhibit glioma cell migration and invasion by regulating the EMT process.

### hsa_circ_0088732 Inhibited Apoptosis and Accelerated the Migration, Invasion, and EMT of Glioma Cells via miR-661 and RAB3D

**Figure 9:**
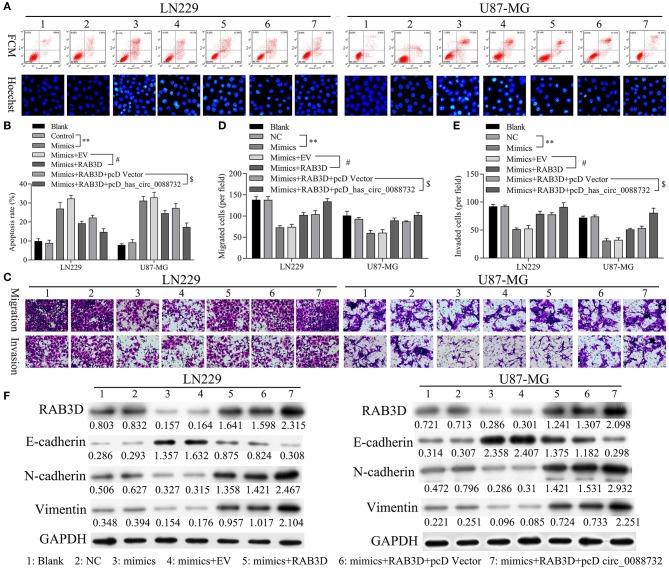
hsa_circ_0088732 inhibited apoptosis and accelerated the migration, invasion, and EMT of glioma cells via miR-661 and RAB3D. LN229 and U87-MG cells were transfected with miR-661 mimics, RAB3D, and hsa_circ_0088732, respectively. The cell apoptosis rates **(A,B)** and numbers of migrated **(C,D)** and invaded **(C,E)** cells were assessed (^**^P < 0.01 vs. control group; ^#^*P* < 0.05 vs. mimics + EV group; ^*$*^*P* < 0.05 vs. mimics + RAB3D + pcDNA vector group). EV, overexpression vector; pcD, pcDNA3.1 plasmid; circ, hsa_circ_0088732. Western blot assays were performed to determine the levels of E-cadherin, N-cadherin, and vimentin expression in the transfected LN229 and U87-MG cells **(F)**.

Rescue experiments were performed to verify that hsa_circ_0088732 could regulate apoptosis, migration, and invasion through its effects on miR-661 and RAB3D in LN229 and U87-MG cells co-transfected with miR-661 mimics, RAB3D or hsa_circ_0088732. Results of flow cytometry and Hoechst 33258 staining studies showed that miR-661 promoted glioma cell apoptosis, while transfection of RAB3D partially rescued the apoptosis mediated by miR-661 mimics; furthermore, hsa_circ_0088732 further inhibited the glioma cell apoptosis mediated by miR-661 mimics and RAB3D (*P* < 0.05, *P* < 0.01, Figures 9A,B). Results of Transwell assays showed that miR-661 suppressed glioma cell migration and invasion, while transfection of RAB3D partially rescued the migration and invasion mediated by miR-661 mimics. Additionally, hsa_circ_0088732 further accelerated the glioma cell migration and invasion mediated by miR-661 mimics and RAB3D, suggesting that the combined effects of hsa_circ_0088732 and RAB3D could rescue the migration and invasion mediated by miR-661 mimics (*P* < 0.05, *P* < 0.01, Figures 9C–E). Therefore, we demonstrated that the effects of hsa_circ_0088732 on glioma cell apoptosis, migration, and invasion were partially due to miR-661 and RAB3D. Our results also proved that miR-661 mimics could inhibit N-cadherin and vimentin expression and promote E-cadherin expression, while transfection of RAB3D rescued the N-cadherin, vimentin, and E-cadherin expression-mediated by miR-661 mimics. Finally, hsa_circ_0088732 further increased N-cadherin and vimentin expression, and decreased the E-cadherin expression mediated by miR-661 mimics and RAB3D (Figure 9F).

### Knockdown of hsa_circ_0088732 Suppressed Glioma Growth *in vivo*

**Figure 10:**
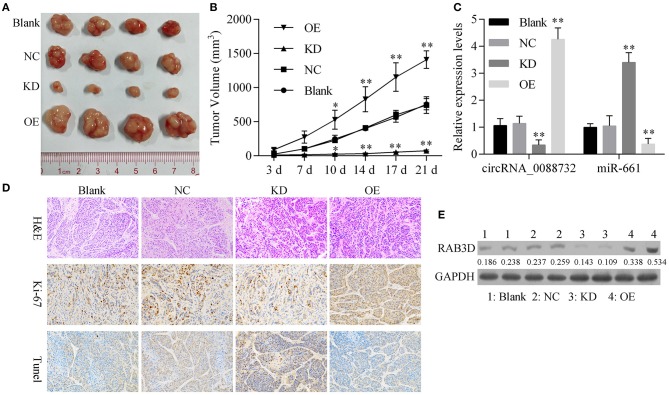
Knockdown of hsa_circ_0088732 suppressed glioma growth *in vivo*. **(A,B)** Gross glioma tumor samples were taken from nude mice at 21 days after injection of LN229 cells (^*^P < 0.05, ^**^P < 0.01). **(C)** qRT-PCR was used to analyze hsa_circ_0088732 and miR-661 expression (^**^P < 0.01). **(D)** Representative images of H&E, Ki-67, and Tunel staining assays. **(E)** Western blot analysis of RAB3D expression. NC: negative control, KD: hsa_circ_0088732 siRNAs, OE: hsa_circ_0088732 overexpression plasmids.

**Figure 11:**
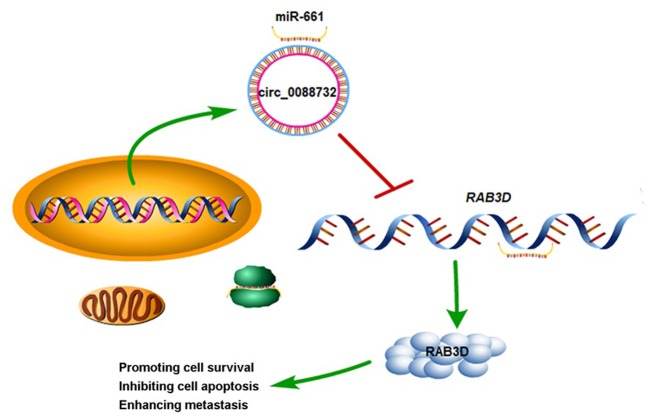
A schematic diagram of the hsa_circ_0088732/miR-661/RAB3D axis in glioma. hsa_circ_0088732 was formed by the *Lcn2* gene by cyclization, hsa_circ_0088732 negatively regulated miR-661, and *RAB3D* was a target gene of miR-661.

To further confirm all the above *in vitro* results showing that knockdown of hsa_circ_0088732 suppressed glioma cell proliferation and metastasis by regulating the miR-661/RAB3D axis, we generated four groups of LN229 cells transfected with (1) transfection reagent (Blank), (2) NC, (3) hsa_circ_0088732 siRNA or (4) hsa_circ_0088732 overexpression plasmids. We then inoculated the cells (1 × 10^6^) into the right axilla of each nude mouse. After 21 days, we found that the mice inoculated with hsa_circ_0088732 siRNA-transfected LN229 cells had smaller tumor sizes than mice in the blank group, and the mice inoculated with LN229 cells transfected with hsa_circ_0088732 overexpression plasmids had larger tumor sizes than mice in the blank group (Figures 10A,B). qPCR results showed that hsa_circ_0088732 levels were decreased and miR-661 levels were increased in the hsa_circ_0088732 siRNA group, while hsa_circ_0088732 expression was increased and miR-661 expression was decreased in the hsa_circ_0088732 overexpression group (Figure 10C). In addition, Ki-67 staining and Tunel assay results showed that knockdown of hsa_circ_0088732 suppressed tumor growth and induced apoptosis, while overexpression of hsa_circ_0088732 promoted tumor growth and inhibited cell apoptosis (Figure 10D). Moreover, western blot results showed that RAB3D expression was significantly decreased in the hsa_circ_0088732 siRNA group and increased in the hsa_circ_0088732 overexpression group (Figure 10E). When taken together, these results confirmed that knockdown of hsa_circ_0088732 suppressed glioma growth *in vivo*. Overall, our study suggested that hsa_circ_0088732 was formed by the *Lcn2* gene by cyclization, hsa_circ_0088732 negatively regulated miR-661, and *RAB3D* was a target gene of miR-661 (Figure 11).

## Discussion

Numerous studies have reported the abnormal expression of various ncRNAs, and particularly miRNAs and lncRNAs found in a variety of cancers. Most of those studies concentrated on the epigenetic regulation of cancer progression (47, 48). Recent studies have suggested that most miRNAs and circRNAs might play regulatory roles in the development and occurrence of glioma (49, 50). However, whether circRNAs have momentous effects on glioma development is far from clear. *hsa_circ_0088732* is located on chr9:130914461-130915734 with a 1273bp genomic length, and is formed by the *Lcn2* gene with the best transcript (NM_005564). Our study is the first report concerning the function and mechanism of hsa_circ_0088732 in glioma. Our results revealed that hsa_circ_0088732 is highly expressed in glioma, and that knockdown of hsa_circ_0088732 can facilitate glioma cell apoptosis *in vitro* and *in vivo*, and also inhibit glioma cell migration and invasion. In addition, we verified that knockdown of hsa_circ_0088732 reduced N-cadherin and vimentin expression, and induced E-cadherin expression. According to previous studies, the EMT is associated with a decreased expression of epithelial markers (E-cadherin), and an increased expression of mesenchymal markers (vimentin and N-cadherin) (51, 52). The EMT is a major biological process by which various malignant tumor cells migrate and invade, and also a key step in the invasion and metastasis of tumor cells (53). Our study is also the first to confirm that knockdown of hsa_circ_0088732 suppresses the EMT process in glioma cells.

In recent years, studies have revealed that circRNAs play a crucial role in regulating gene expression by serving as competing endogenous RNAs (ceRNAs). In addition, with the decreased polymorphism of miRNA response elements (MREs), exonic circRNAs become more effective as miRNA sponges. For example, the circular RNA ciRS-7 (Cdr1as) contains 74 binding sites for miR-7, which has been extensively isolated from CDR1, and makes it an effective “miR-7 sponge” (34); another circRNA designated as sex-determining region Y (Sry) was also verified to act as a sponge for miR-138 (35). In our study, we confirmed that hsa_circ_0088732 serves as a sponge for miR-661. In addition, we demonstrated that miR-661 was expressed at low levels in glioma, and negatively correlated with hsa_circ_0088732 expression. With regard to function, our results verified that knockdown of miR-661 suppressed cell apoptosis and promoted glioma cell migration, invasion, and the EMT. We also showed that a miR-661 inhibitor could attenuate the increase in glioma cell apoptosis induced by hsa_circ_0088732 knockdown, suggesting that hsa_circ_0088732 contributes to glioma progression by targeting miR-661. Previous studies have also shown that numerous miRNAs can regulate the EMT process, including members of the miRNA-200 family, miRNA-141, and miRNA-429 (54–58). Furthermore, miRNAs were shown to play vital roles in the development and occurrence of glioma by regulating the EMT (59, 60). We also proved the effect of miR-661 on the EMT process in glioma.

Scientific studies have proven that stable mRNA transcripts contain several RNA-binding sites or MREs, which might also serve as miRNA sponges. This might allow miRNA to regulate gene expression at the post-transcription level by binding to the 3′-untranslated regions (3′-UTRs) (61). In our study, we found that RAB3D was significantly upregulated in glioma, and negatively correlated with miR-661 expression. Additionally, a bioinformatics analysis showed there was a binding site for miR-661 in the 3′UTR of RAB3D mRNA, and luciferase reporter assays confirmed that *RAB3D* was a target gene of miR-661. *RAB3D* is a member of the RAS gene family of proto-oncogenes, and the RAB3D protein is a guanosine triphosphate (GTP) binding protein (62). RAB3D involvement has been implicated in multiple regulatory processes, such as vesicle transport, protein secretion, and signal transduction (63, 64). It was reported that RAB3D is highly expressed in tumor tissues and cells, and promotes the migration and invasion of tumor cells (65, 66). In our study, we found that RAB3D can act as an oncogene in glioma, and hsa_circ_0088732 can regulate RAB3D expression by sponging miR-661.

## Conclusions

We suggest that hsa_circ_0088732 suppresses apoptosis and promotes the migration and invasion of glioma cells via the miR-661/RAB3D axis, whose function may involve molecular targets useful for diagnosing and treating glioma. This study highlights the diagnostic and therapeutic potential of the hsa_circ_0088732/miR-661/RAB3D axis in glioma, and provides a theoretical mechanism for the development and occurrence of glioma.

## Data Availability Statement

The raw data supporting the conclusions of this article will be made available by the authors, without undue reservation, to any qualified researcher.

## Ethics Statement

The animal study was reviewed and approved by Ethics Committee of the Affiliated Shantou Hospital of Sun Yat-sen University.

## Author Contributions

YK conceived the method and the experiments, and supervised the project. TJ, YLiu, ZX, and YZ performed experiments. TJ, ML, YLi, HH, and JL analyzed the results. TJ, ML, YLiu, and YZ wrote the first draft. YK revised the manuscript.

### Conflict of Interest

The authors declare that the research was conducted in the absence of any commercial or financial relationships that could be construed as a potential conflict of interest.

## Supplementary Material

Figure S1 The detection of migration and invasion abilities of A172, LN229, U87-MG, and U251 cell lines.

Figure S2 The detection of migration and invasion abilities of HEB and A172 cell lines transfected with hsa_circ_0088732 overexpression plasmids. ^**^*P* < 0.01.
