# Supplementary figures and images for: Lcn2-derived Circular RNA (hsa_circ_0088732) Inhibits Cell Apoptosis and Promotes EMT in Glioma via the miR-661/RAB3D Axis
Source: Front Oncol. 2020 Feb 21;10:170. doi: 10.3389/fonc.2020.00170 (PMC7047435; doi:10.3389/fonc.2020.00170)

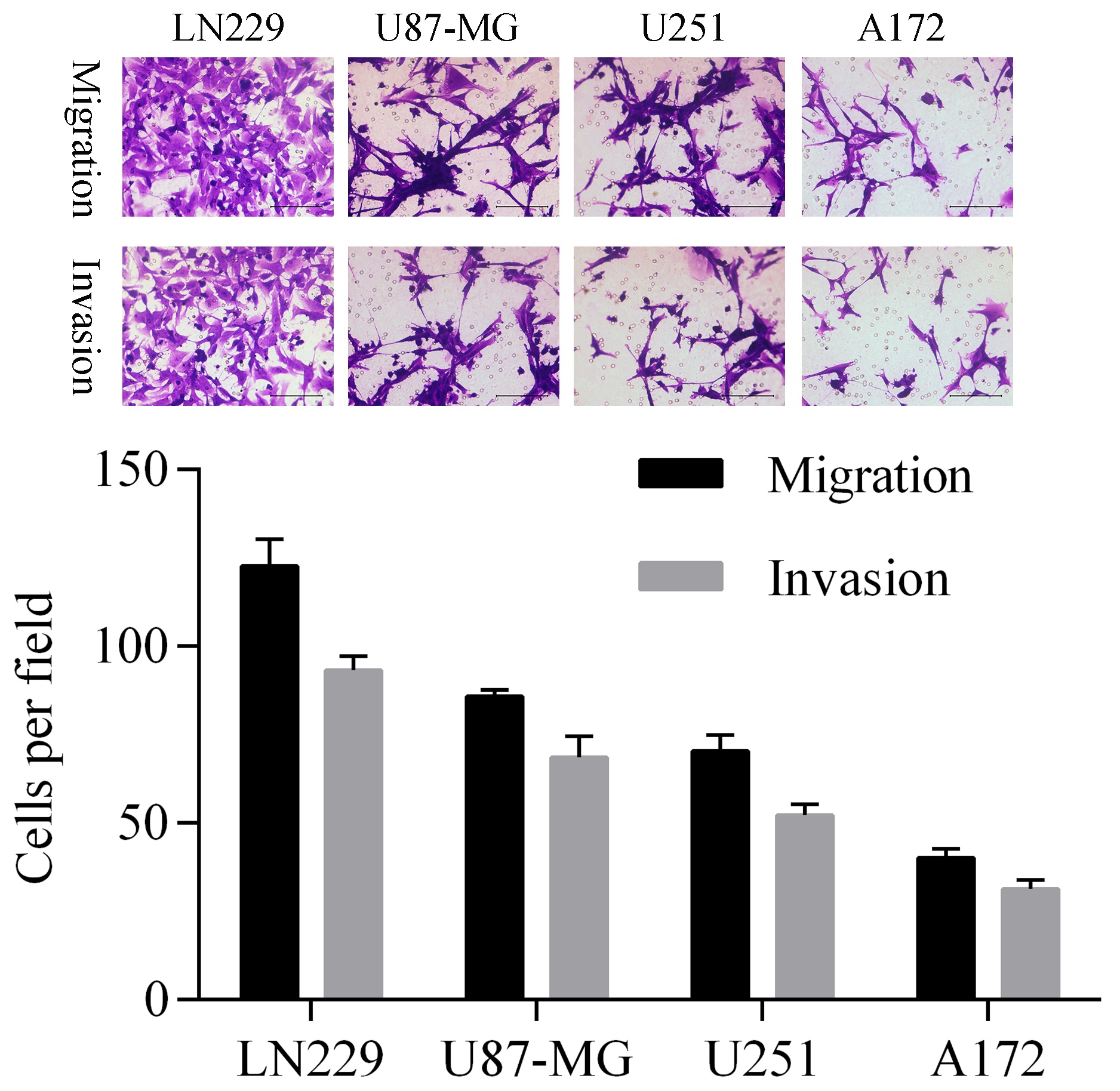

Supplement: Figure S1 — The detection of migration and invasion abilities of A172, LN229, U87-MG, and U251 cell lines. [file Image_1.JPEG]

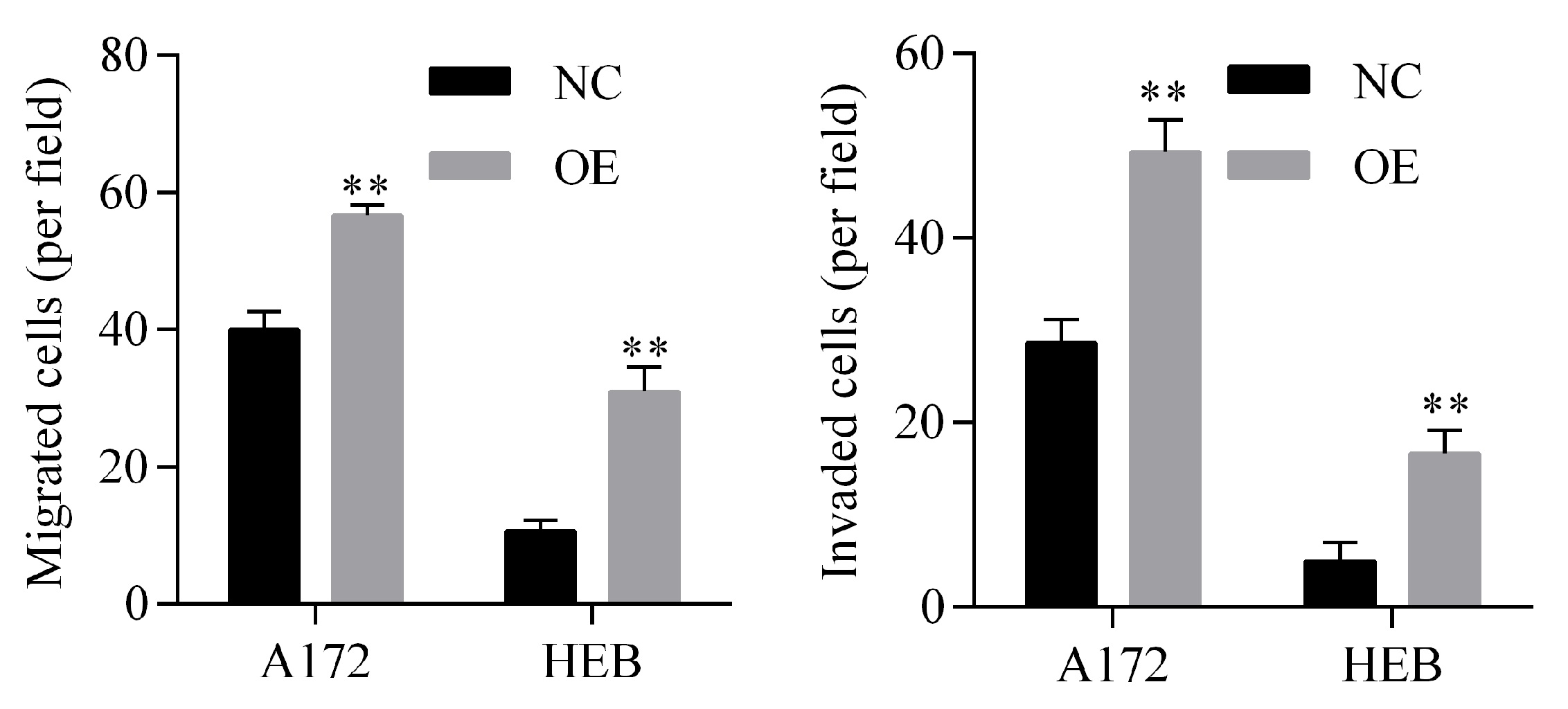

Supplement: Figure S2 — The detection of migration and invasion abilities of HEB and A172 cell lines transfected with hsa_circ_0088732 overexpression plasmids. **P < 0.01. [file Image_2.JPEG]
